# The Effect of Allograft Transplantation of Sertoli Cell on Expression of NF-кB, Bax Proteins, and Ischemic Tolerance in Rats with Focal Cerebral Ischemia

**DOI:** 10.22037/ijpr.2020.15574.13189

**Published:** 2020

**Authors:** Roya Hosseini, Mohammad Reza Bigdeli, Sepideh Khaksar, Abbas Aliaghaei

**Affiliations:** a *Department of Physiology, Faculty of Life Sciences and Biotechnology, Shahid Beheshti University, Tehran, Iran.*; b *Institute for Cognitive and Brain Sciences, Shahid Beheshti University, Tehran, Iran. *; c *Department of Plant Sciences, Faculty of Biological Sciences, Alzahra University, Tehran, Iran. *; d *Department of Anatomy and Cell Biology, School of Medicine, Shahid Beheshti University of Medical Sciences, Tehran, Iran.*

**Keywords:** Cell transplantation, Brain ischemia, Infarction, Blood-brain barrier, Edema, Inflammation, Apoptosis

## Abstract

One of the newest methods to reduce cerebral ischemia damages is cell therapy. The aim of this study is to evaluate the effect of Sertoli cell transplantation on ischemia-induced injuries in animal models of stroke. Rats were divided into four groups: transplant+ischemia, ischemia, sham, and control. Sertoli cells were separated from the other testis of rats and cultured. Unilateral Sertoli cell transplantation was performed in the right striatum by using stereotaxic surgery. For induction of brain ischemia, middle cerebral artery occlusion surgery was used 14 days after transplantation. By using western blotting method, expression of nuclear factor kappa (NF-кB) and Bax were evaluated. In this study, a remarkable decrease in neurological deficits, infection, blood-brain barrier permeability, and brain edema was observed in the cell transplant recipient group in comparison with the ischemia group. Probably, a reduction in inflammation (NF-кB factor) and apoptosis (Bax) following injection of Sertoli cells result in amelioration of ischemic damages induced by MCAO surgery.

## Introduction

Stroke is the second and third cause of death, after cancer and cardiovascular diseases, in developing and industrialized countries, respectively. Also, it is possible that compulsive ischemia occurs during surgery in some diseases such as aneurysm and aortic disorder, in which the patients deal with signs of stroke and different degrees of physical disability (which is sometimes irreversible) after surgery (1, 2). So, increased brain cellular tolerance to ischemia and the reduction of cell death has been considered as a clinical goal in these diseases. Therefore, achieving this can be a big consequence in this direction. 

In fact, cerebral ischemia is the result of impaired blood supply to part of the brain tissue due to the obstruction of the vessels by a blood clot or rupture of the vessels supplying the brain tissue (3). In the absence of blood supply and oxygen, a cascade of events is created: Anaerobic metabolism, imbalance of ionic gradients along the cell membrane, increased intracellular osmolality, cytotoxic edema, increased activity of matrix metalloproteins, breakdown of the blood-brain barrier, entry of leukocytes into the brain tissue, and finally vasogenic edema (4-6). In addition, ischemia causes an immune response triggering a cascade of events that eventually leads to inflammation and necrotic death of neurons. Clinical studies have shown that there is a close relationship between higher inflammatory markers and the risk of cerebral ischemia. Despite extensive studies conducted on the biology of stroke, there are few effective therapies for this condition (7). 

NF-KB (kappa-light chain nuclear factor) in activated B cells is a protein complex that controls DNA transcription. This factor regulates the expression of genes involved in inflammation, immune responses and, most of all, apoptosis. Many of these genes are also active in damage caused by reperfusion blood flow in stroke (7). Bax (Bcl-2 Associated X Protein) at the onset of apoptosis, undergoes a structural change and attaches to the membrane of organelles, especially mitochondria. The Bax protein opens the mitochondrial voltage anion channel, which leads to loss of membrane potential. The Bax protein also forms a pore in the outer mucosal mitochondria called MAC, which ultimately releases cytochrome C, and other pro-apoptotic proteins from mitochondria and activates Caspase (8).

In the present study, pretreatment with Sertoli cells is used in order to induce an adaptive-protective response and increase the patient’s resistance to future severe ischemic injury. Sertoli cells are located in the testicles, Sertoli cells provide an environment for the development of sex cells that trigger an immune response (9, 10). Moreover, Sertoli cells express some growth factors, including insulin-like growth factor (IGF), alpha and beta transforming growth factor (TGF), glial cell-derived neurotrophic factor (GDNF), fibroblast growth factor (FGF), vascular endothelial growth factor (VEGF), and Ciliary neurotrophic factor (CNTF) (9-11). In addition, to protect themselves against oxidative stress, Sertoli cells secrete high levels of antioxidant enzymes, including Superoxide dismutase (SOD), Glutathione S-transferase (GST), and Glutathione reductase (GR) (12, 13). Based on the evidence, these cells are expected to be able to inhibit apoptosis, inflammation, free radicals, destruction of the blood-brain barrier, and edema.

On the other hand, the distinctive characteristic of Sertoli cells, compared to other somatic cells and stem cells, is the inhibition of the immune system which prevents the transplant rejection by the recipient. That is why these cells were selected to be studied in this research. Sertoli cells apply four mechanisms to suppress the immune system: first, Sertoli cells come together by creating tight connections and forming an immunological barrier around the nerve cells that prevent the immune system from affecting these cells. Second, production of agents that inhibit the expression of Interleukin 2 (IL2) and proliferation of T-cells (it is noteworthy that IL2 stimulates the proliferation and accumulation of T-cells in the affected area by launching the signaling path and triggering the severe reaction of the immune system). Third, non-expression of the major histocompatibility complex (MHC) surface antigens which are present on the cell surface of all tissues and are identified by the immune system. Fourth, expression of Fas ligand (FasL) occurs by these cells. These legends attach to Fas molecules on the surface of lymphocytes and induce apoptosis in them (10). 

In fact, the other potential properties of Sertoli cells are the ability of these cells in the secretion of antioxidant and cytoprotective factors and functional support for the other cells as nursing cells. Furthermore, Sertoli cells promote them as a nerve protector to support the cells of the ischemic region and its surrounding area (11). The present research was designed to study the effect of pretreatment of Sertoli cells on serious damages caused by cerebral ischemia, including neurological defects, infarction, the blood-brain barrier permeability, edema, inflammation, and apoptosis, in the animal model of cerebral ischemia. 

## Experimental

*Animals and group assignment*

In this study, 84 Wistar adult male rats, weighted 250-300 g, were selected randomly as subjects and assigned to 4 groups of sham, ischemia (given cell transplantation without ischemia), ischemia (given ischemia without cell transplantation), and transplant+ischemia (given cell transplantation and undergone ischemic surgery). In rats of the transplant recipient group, Sertoli cells were obtained from the other testicular tissue (from another rat). This group was subdivided into 4 subgroups in order to study infarct volume (n = 7), the permeability of the blood-brain barrier (n = 7), edema (n = 7), and the expression levels of NF-kB and Bax proteins (n = 7). In the ischemia group, the effect of cerebral ischemia, without cell transplantation, was assayed on infarct volume (n = 7), the permeability of the blood-brain barrier (n = 7), edema (n = 7), and expression levels of NF-kB and Bax proteins (n = 7). In the sham group, the effect of the stress of surgery and injection of Sertoli cell culture medium without ischemia and transplantation was evaluated (3 subgroups: permeability of the blood-brain barrier (n = 7), edema (n = 7), expression levels of NF-kB and Bax proteins (n = 7)). Due to the lack of ischemic induction, the infarction volume was not evaluated in the sham group. The effect of Sertoli cell transplantation without ischemia was considered in the control group (1 subgroup: expression levels of NF-kB and Bax proteins (n = 7)) (Table 1).

The sham group is a non-ischemic group. This group was considered for the study of the effect of stress resulting from surgery and the effect of the infusion of Sertoli sulfate into the brain. Therefore, neurological defects and the other items should be investigated for comparison between sham, ischemia, and transplant+ischemia groups. But the control group was only considered as an evidence for the expression of inflammatory and apoptotic proteins.

In the transplant+ischemia group, the rats were subjected to 60 min of middle cerebral artery occlusion (MCAO) surgery 14 days after the injection of Sertoli cells. Twenty-four hours later, neurologic deficits, brain edema, infarct volume, and blood-brain barrier (BBB) integrity were evaluated.

*Ethical Statement*

This study was conducted in accordance with the rules of the National Institutes of Health and Care Guidance and the use of the Animal Laboratory (NIH Publications) revised in 2011, and the ethics committee of Shahid Beheshti University (No. 932,696). In this study, as little as possible, the animals have been used. 

Death rates for groups: Control = 0, Sham = 13.3%, Ischemia = 33.3%, Transplant recipient = 20%.

*Isolation and culture of Sertoli cells*

Under approval by the animal care committee of Shahid Beheshti University of medical sciences, Wistar male rats (100-120 g) (n = 3) were killed under deep anesthesia (chloral hydrate, 800 mg/kg) and the testicles were removed. The tunica albuginea was removed from the testis and the tissue was sequentially digested first with trypsin (0.25%) (Gibco, USA) for 15 min and then with collagenase (0.1%) for 15 min at 37 °C. Afterward, fetal bovine serum (FBS) (Gibco, USA) was added and after centrifugation of the solution, the pellet was transferred to a culture media containing Dulbecco’s Modified Eagle’s Medium (DMEM/F12) (Gibco, USA), FBS (10%) and antibiotic. After 48 h, to remove the debris and red blood cells, the culture medium was changed (9). 

*Immunocytoflouresent*

Sertoli cells were cultured in 24-well plate and fixed by 4% paraformaldehyde (PFA) (Merk, Germany). After washing with Phosphate Buffered Saline (PBS), cells were permeabilized with Triton Χ-100 (Sigma Aldrich, USA). Then, cells were incubated with goat normal serum followed by overnight incubation with the primary antibody against GATA4 (a member of the GATA transcription factor family) (Abcam, USA) at 4 °C. The fluorescent secondary antibody (goat anti-rabbit conjugated FITC (Abcam, USA)) was applied after washing with PBS. For visualization of the nuclei, the cells were stained with DAPI (Santa Cruz Biotechnology, USA). Preparations were examined under the fluorescent microscope (14). 

*Sertoli cell transplantation*

Unilateral Sertoli cell transplantation was performed in the right striatum. The striatum is associated with the cerebral cortex and the secreted trophic factors of Sertoli cells are absorbed by the striatum and sent to the cerebral cortex in the form of retrograde. The Sertoli cells were maintained alive in suspension using a 2 µL DMEM aliquot, stored on ice during the surgery procedure. After anesthetizing, the animals were unilaterally transplanted with 500.000 Sertoli cells in the right striatum labeled with DiI using a 5 µL Hamilton microsyringe placed at the following coordinates, relative to Bregma: +0.5 mm AP; −2.6 mm ML; −5 mm DV. Sertoli cells were transplanted in the part of the striatum (medio-posterior part) which was devoid of disruption of the extracellular matrix, in order to maximize graft survival. Based on the articles and also because the brain tissue does not have the capacity for more cells if more cells are injected, some injected cells are rejected (15, 16). 

*Detection of the Sertoli cells after transplantation*

Before injection of the Sertoli cells, they were labeled with DiI (incubated with DiI, 5 μg/mL for 20 min before injection) and Hoechst (Sigma Aldrich, USA) (incubated with Hoechst, 4 μg/mL for one hour before injection) staining. After 14 Days, the rat was killed and the brain was prepared to determine the survival and distribution of the transplanted cells. Detection of these cells were carried out by fluorescing microscopy.

*Induction of cerebral ischemia by the MCAO method*

Fourteen days after the recovery (Time to show trophic effects of Sertoli cells), the rats were anesthetized to undergo an intercostal artery occlusion surgery. For this purpose, a nylon suture (3-0) was inserted into the internal carotid artery through the trunk of the common carotid artery to reach the anterior carotid artery. As a result of the insertion of the suture and blockage of the arterial blood flow pathways, the blood flow from each side was obstructed to the middle cerebral artery (MCA). After 60 min of ischemia, the blood flow was restored. The body temperature was measured through a rectum digital thermometer and maintained at 37 °C using a thermal pad (17).

*Neurologic deficits Score*

Twenty-four hours after the induction of ischemia, behavioral tests were performed on the rats to evaluate neurologic deficits. Neurological tests were conducted in five categories of Raise the Tail, Sensory Function, Motor Function, Beam Test and reflex activity (the minimum and maximum scores on these tests were 0 and 18, respectively) (18).

*Infarct volume*

After performing neurological tests and killing the rats, their brain was removed and kept in cold saline at 4 °C for 5 min. Then, coronal brain slides with a thickness of 2 mm were prepared using the brain matrix. The brain slices were put in 2% TTC, 2,3,5-triphenyl tetrazolium chloride solution, solution (Merck, Germany) at 37 °C for 15 min to be stained. Then, some photos were taken from these slides using a digital camera (Canon EOS 500D digital). Finally, the area of the ischemic site in each slide was measured using Image J (Version 1.50) and calculated using the Swanson method based on the following equation. In this part, infarct volume was analyzed in the whole hemisphere, cortex, piriform cortex-amygdala, and striatum separately. The brain areas were identified using the Rat Brain Atlas of Paxinos and Watson (19). 

The corrected volume of damaged area = Left Hemisphere Volume – (Right Hemisphere Volume – Damaged Area Volume).

*The permeability of the blood-brain barrier*

The integrity of the blood-brain barrier was evaluated by measuring the amount of Evans Blue dye (EBD) (Sigma Chemicals, USA) extravasation. First, 30 min after the onset of ischemia, the rats were treated with 4 mL of 2% Evans Blue per body weight intravenously. Twenty-four hours after the induction of ischemia, the rats were anesthetized and intravascular Evans blue was replaced with saline using the transcardial method. Then the brain was removed and its different parts (cortex, piriform cortex-amygdala, and striatum) were separated in each hemisphere and weighted. To measure the EBD extravasation, the brain tissue was homogenized in phosphate buffer and 60% trichloroacetic acid was added to the solution to precipitate the protein. Then, the microtubes were vortexed for 2 min and kept in a fridge at 4 °C for 30 min. In the next stage, the samples were centrifuged at 1000 rpm for 30 min. Finally, the EBD absorbance in the supernatant was measured at 610 nm wavelength by a spectrophotometer (Perkin-Elmer, Illinois, USA) and its concentration was calculated according to the standard curve and expressed as μg/g of brain tissue (20, 21).

*The water content of the tissue*

After decapitation of rats, their brain was removed and the cerebellum, pons, and olfactory bulbs were separated. Then, the wet weight (WW) of different parts of the brain (cortex, piriform cortex-amygdala, and striatum) was assessed. After drying them in an oven at 120 °C for 24 h, their dry weight (DW) was also measured. Finally, the water content of the brain was calculated using the following Equation (20): [(WW−DW)/WW] × 100

*Western blot*

The studied brain zones (cortex, piriform cortex-amygdala, and striatum) for each group (sham, control, ischemia, and transplant+ischemia) were identified and isolated using the Rat Brain Atlas of Paxinos and Watson. Equal volumes of obtaining samples to prepare the protein extract were homogenized and centrifuged with a lysis solution (Tris-Hcl_SDS_EDTA_NaCl_Sodium Deoxy cholate_Protease inhibitor cocktail_NP40 (0.1%)) and the supernatant was achieved. Supernatants were transferred to the gel using the electrophoresis gel. Proteins were separated by SDS-PAGE (10% gel) and then transferred to the PVDF membrane (Millipore) using a Western tank containing a transfer buffer (Tris_Glycine_Methanol 20%_Distilled Water). Blocking of blots was performed by using a blocking solution containing 2% non-fat dry milk in Tris buffer saline in 0.1% Tween 20 at 4 ºC for 75 min. Then, the blots were incubated with rabbit anti-NF-кB polyclonal (1:500 dilution; Santa Cruz), rabbit anti-Bax polyclonal Antibody (1:500 dilution; Santa Cruz, USA) and rabbit anti-β-actin (1:1000 dilution; Santa Cruz) antibodies, followed by goat anti-rabbit secondary antibody (1:500 dilution; Santa Cruz) for 90 min separately. Finally, the desired proteins were identified using advanced chemiluminescence (Enhanced Chemiluminescence, Amersham Biosciences) and film exposure. The signal density of the blots was measured by an image analysis system (ImageJ, version 1.46r).

*Statistical analysis*

Data analysis was performed in SPSS (v22.0). Data from neurologic deficits were analyzed using Kruskal-Wallis followed by the Dunn’s test and the statistical analysis of data obtained from infarct volume, was done by non-parametric Mann-Whitney U test (SPSS v22.0). Moreover, brain edema, blood-brain barrier permeability, and expression of NF-kB and Bax proteins were compared using two-way analysis of variance (ANOVA) (SPSS v22.0 *post-hoc* LSD). The results were reported as mean ± SEM and the level of significance was determined to be (*P *< 0.05).

## Results

*Sertoli cells showed a fibroblast-like phenotype*

**Figure 1:**
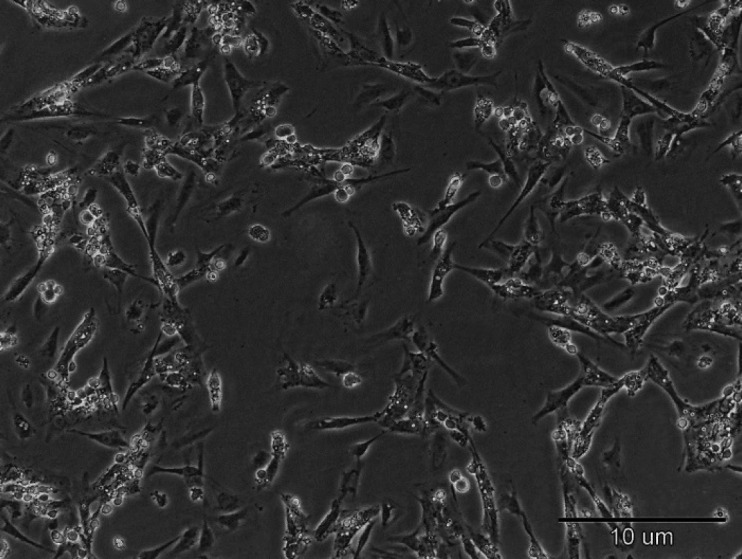
Phase contrast microscopy analysis after 14 days of culture of Sertoli cells shows Sertoli cells appeared fibroblast-like cells

Fourteen days after we cultured Sertoli cells, fibroblast-like cells appeared in culture dishes. The cells grew at high speed and rapidly covered the surface. This subject exhibits the ability of Sertoli cells in proliferation and activation (Figure 1).

*Immunocytoflouresent proves the presence of the Sertoli cells in the testis-derived samples*

**Figure 2:**
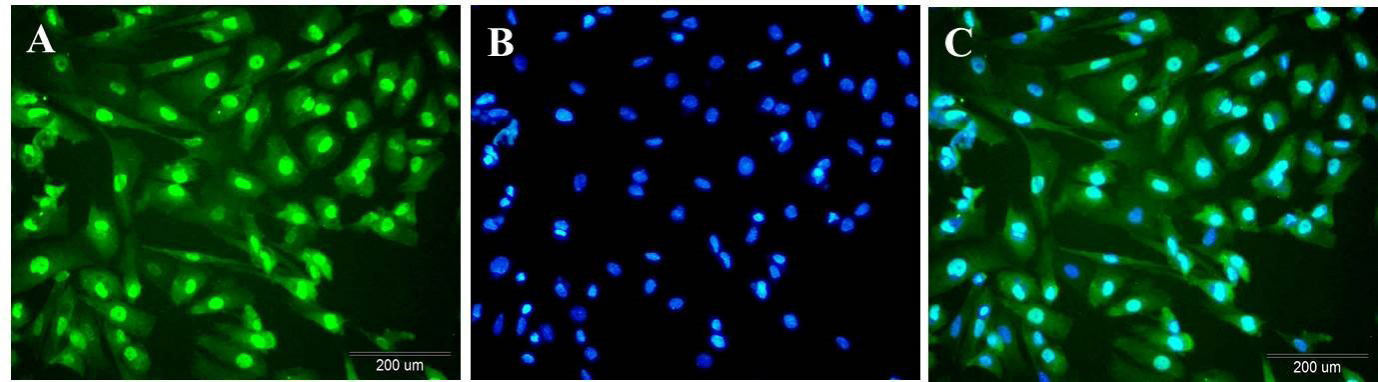
Immunocytoflouresent of Sertoli cells against anti GATA4 demonstrates testis-derived cells are Sertoli cells. (A) anti-GATA4-stained Sertoli cells are in green; (B) Blue nuclei staining with Hoechst; (C) Merge of two pictures

Immunocytochemistry analysis indicated that Sertoli cells were immunopositive for GATA4 that is expressed in Sertoli cells. These cells are in green (Figure 2A). Their nucleus was becoming blue with Hoechst (Figure 2B). Hoechst -stained Sertoli cells were revealed in green color with blue nuclei (Figure 2C).

*Sertoli cells survived in the brain after transplantation*

**Figure 3:**
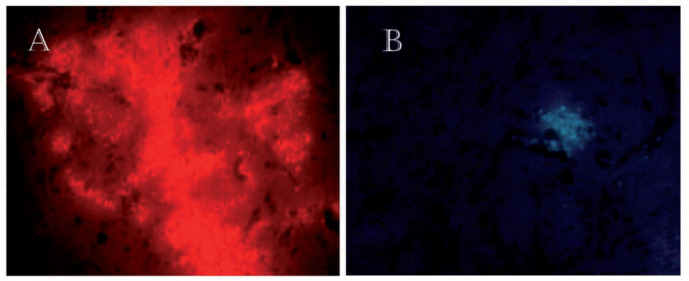
To ensure the survival of transplanted Sertoli cells in the striatum, 14 days after the injection, were tracked by fluorescence microscopy. (A) The injected cells into the striatum were labeled with DiI. × 40. (B) The transplanted cells were stained with Hoechst and injected into the striatum. × 20. These figures approve survival of Sertoli cells 14 days after transplantation

Fourteen days after the injection of Sertoli cells into the striatum of rats, the results showed that these cells were surviving in the brain. The Sertoli cells were labeled with DiI and Hoechst (Figure 3).

*Pretreatment with Sertoli cell transplantation reduced the score of neurologic deficits in the transplant recipient group*

**Figure 4:**
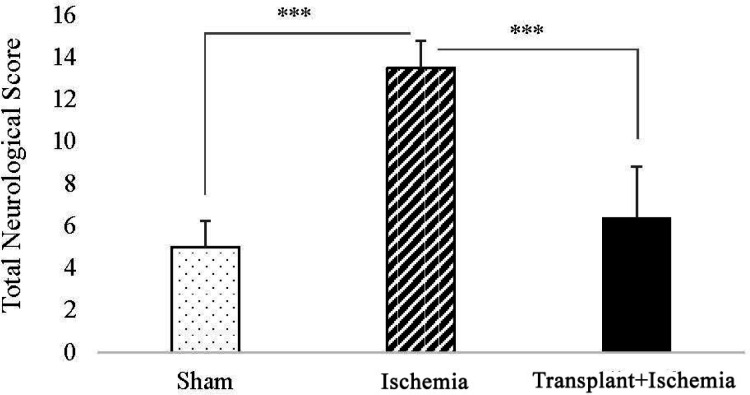
Effect of Sertoli cell transplantation on total neurological deficits at 24 h after ischemia induction in rats. Values are expressed as the mean ± SEM (n = 10). *P *˂ 0.05 considered as significant. (^*^P < 0.05, ^**^P < 0.01, ^***^P < 0.001) (Nonparametric Kruskal- Wallis analysis).

In the comparison between the sham and ischemia groups, an increased total score of neurologic deficits and all tests separately was observed in the ischemia group (*P < *0.001). In addition, analysis of neurologic deficits scores showed a reduction in the transplant+ischemia group compared to the ischemia (*P < *0.001) (Figure 4) (Table 2).

*Sertoli cells attenuated infarction volume caused by cerebral ischemia in the transplant+ischemia group*

**Figure 5:**
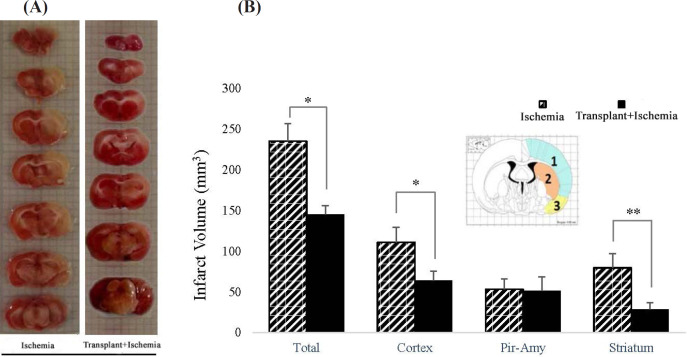
(A) Left: coronal brain cutting of rat brain stained with TTC in ischemia group; Right: coronal brain cutting of rat brain stained with TTC in transplant+ischemia group. Infarcted zone was shown with colorless or white regions. (B) Effect of Sertoli cell transplantation on infarct volume in total of the hemisphere, cortex (blue: 1), striatum (orange: 2), and piriform-amygdala cortex (yellow: 3) 24 h after MCAO surgery. Each column represents mean ± SEM (n = 7). *P *˂ 0.05 considered as significant. (^*^P < 0.05, ^**^P < 0.01, ^***^P < 0.001) (Mann-Whitney U test).

In the ischemia group, the total volume of tissue infarction was equal to 221.3 ± 8.7 mm^3^. The results showed that Sertoli cell transplantation reduced the total volume of infarction (166.82 ± 8.7 mm^3^) (*P *= 0.03). Moreover, the decrease in the infarction was observed in the cortex of the Sertoli cell transplant recipient group (64.07 ± 14.5 mm^3^) compared to the ischemia (102.59 ± 20.9 mm^3^) (*P *= 0.03). Reduced infarct volume in the striatum in the transplant+ischemia group (21.82 ± 9.34 mm^3^) was significant compared with the ischemia group (59.2 ± 20.5 mm^3^) (*P *= 0.01). However, no significant difference was observed between the two groups in the piriform cortex-amygdala (Figures 5A and 5B).

*Sertoli cell transplantation increased the integrity of the blood-brain barrier in the ischemic region*

**Figure 6:**
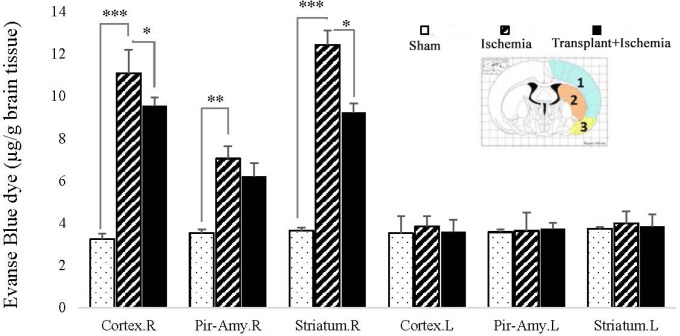
Effect of Sertoli cell transplantation on blood-brain damages in the cortex (blue: 1), striatum (orange: 2), and piriform-amygdala cortex (yellow: 3) 24 hours after MCAO surgery. Each column represents mean ± SEM (n = 7). *P *˂ 0.05 is considered significant. (^*^P < 0.05, ^**^P < 0.01, ^***^P < 0.001) (Two-way ANOVA test).

The findings indicated that the Evans blue extravasation from the blood-brain barrier in the ischemic hemisphere increased in the ischemia group more than the sham group in all three brain regions (*P < *0.001). There was no significant difference between the two hemispheres of the sham group in the presence of EBD. In addition, the concentration of EBD in the left hemisphere of the brain showed no significant difference between the studied groups. This indicates the maintenances of the blood-brain barrier integrity in the left hemisphere of these groups. The concentration of Evans blue dye in the cortex (7.70 ± 0.32) and striatum (7.01 ± 0.27) zones of the right hemisphere in the Sertoli cell transplant recipient group showed a reduction compared to the ischemia (cortex: 9.32 ± 1.19, striatum: 10.31 ± 0.44) (*P *= 0.03, *P *= 0.01). However, Sertoli cell transplantation made no change to the permeability of the blood-brain barrier in the piriform cortex-amygdala. Accordingly, the results confirmed the positive effect of Sertoli cells on the reduction of damage in the transplant recipient group. Furthermore, surgery and injection of Sertoli cell culture into the brain without induction of ischemia had no effect on the blood-brain barrier integrity (Figure 6).

*Sertoli cell transplantation in the transplant+ischemia led to the reduction of the ischemia-induced brain water content*

**Figure 7.**
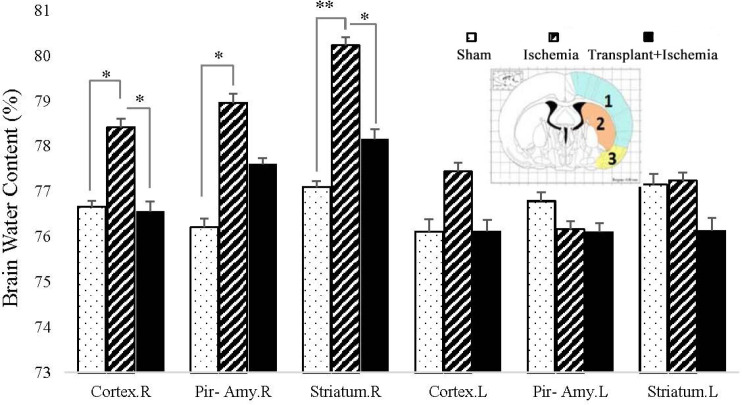
Effect of Sertoli cell transplantation on brain water content in the cortex (blue: 1), striatum (orange: 2), and piriform cortex-amygdala (yellow: 3) 24 h after MCAO surgery. Each column represents mean ± SEM (n = 7). *P *˂ 0.05 is considered significant. (^*^P < 0.05, ^**^P < 0.01, ^***^P < 0.001) (Two-way ANOVA test).

The results expressed that the brain water content in the cortex of the ischemic hemisphere in the transplant recipient group (74.95 ± 0.19%) showed more reduction than the ischemia group (76.82 ± 0.28%) (*P *= 0.03). There was no significant difference between the transplant recipient and ischemia groups in the piriform cortex-amygdala. In addition, Sertoli cell transplantation attenuated the brain water content in the striatum of the transplant+ischemia group (75.04 ± 0.25%) compared to the ischemia (77.01 ± 0.28%) (*P < *0.01). The comparison of the cortex, piriform cortex-amygdala, and striatum between the ischemia and sham groups showed an increase in edema in the ischemia group which indicates the effect of ischemia on increased cerebral edema in the ischemia group (*P *= 0.01, *P *= 0.01, and *P < *0.01) (Figure 7).

*Sertoli cell transplantation presented a decreasing effect on the expression of inflammatory factor (NF-kB) in the transplant+ischemia group*

**Figure 8:**
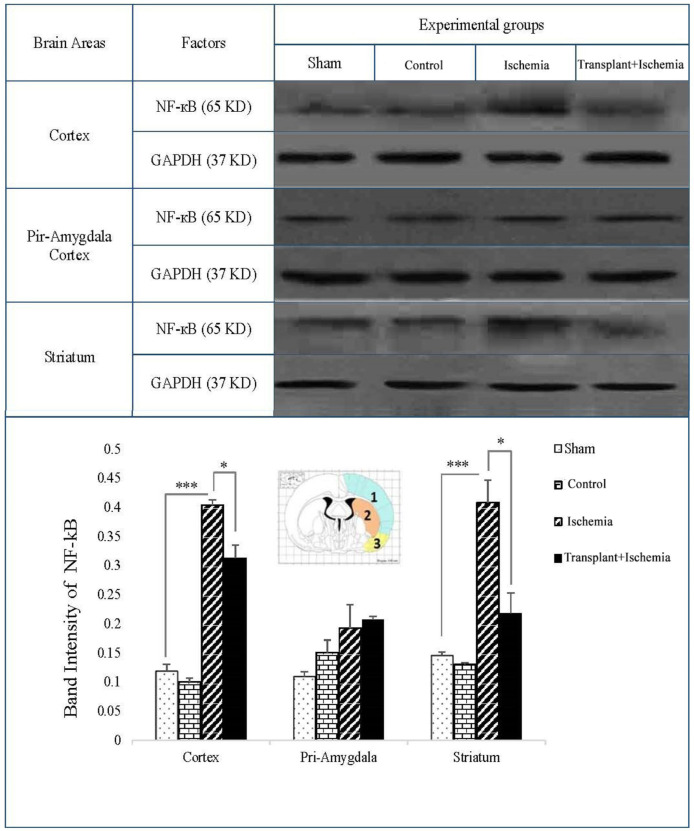
Effect of Sertoli cell transplantation on the expression of NF-kB factor in the cortex (blue: 1), striatum (orange: 2), and piriform-amygdala cortex (yellow: 3). the analysis of NF-kB bands after normalization with GAPDH was performed as a ischemia group loading. Each column represents mean ± SEM (n = 7). (^*^P < 0.05, ^**^P < 0.01, ^***^P < 0.001) (One-way ANOVA test).

The expression of NF-kB in the sham and control groups in three zones of the brain was very low and there was no significant difference between these groups. In the comparison between the three groups, an increase in the expression of NF-kB was observed in the ischemia group compared to the sham in the cortex and striatum (*P < *0.001). Moreover, the observations indicated that the side effects of various factors such as surgical stress, infusion of cell culture medium, and Sertoli cell transplantation without the induction of ischemia have no effect on the expression of the mentioned factors and induction of inflammation. The expression of NF-kB in the cortex of the recipient group showed a reduction compared to the ischemia group (*P *= 0.03). The comparison between transplant+ischemia and ischemia groups in the piriform cortex-amygdala presented no significant difference. In addition, a reduction in the expression of NF-kB protein level of the striatum was observed in the transplant recipient group compared to the ischemia (*P *= 0.01) (Figure 8).

*Sertoli cell transplantation reduced the expression of the pro-apoptotic factor of Bax in the transplant+ischemia group*

**Figure 9:**
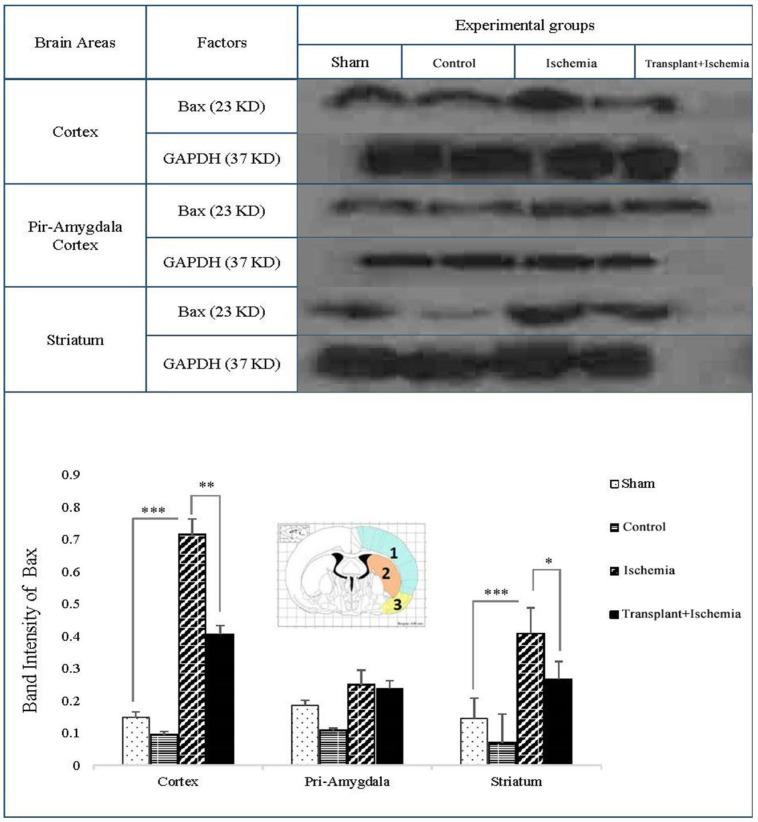
Effect of Sertoli cell transplantation on the expression of Bax factor in the cortex (blue: 1), striatum (orange: 2) and piriform-amygdala cortex (yellow: 3). The analysis of Bax bands after normalization with GAPDH was performed as control loading. Each column represents mean ± SEM (n = 7). (^*^P < 0.05, ^**^P < 0.01, ^***^P < 0.001) (One-way ANOVA test).

One of the key factors in the apoptosis signaling pathway is the Bax factor that the prevention or reduction of it is one of the objectives of cell therapy in stroke studies. Based on these results, the expression of Bax in sham and control group did not show a significant decrease in the cortex, piriform-amygdala, and striatum cortex. Furthermore, the expression of this protein in the ischemia group increased in the ischemia group compared to the sham group in the cortex and striatum areas (*P < *0.001). In the cortex, the Bax expression in the transplant recipient group was also reduced compared to the ischemia group (*P < *0.01). There was no significant change in the expression of the Bax factor in the cortex-parietal-amygdala cortex. In the striatum region, the expression of Bax decreased in the transplant+ischemia group compared to the ischemia group (*P *= 0.03) (Figure 9).

**Table 1:** Assignments and evaluated items in experimental groups. MCAO: middle cerebral artery occlusion; BBB: Blood-Brain Barrier; NF-кB: nuclear factor kappa

**Assignment**	**Control**	**Sham**	**Ischemia**	**Transplant+ischemia**
	**Transplantation** **No MCAO**	**No Transplantation** **No MCAO**	**No Transplantation** **MCAO**	**Transplantation** **MCAO**
Infarction Volume Brain Edema BBB Permeability NF-кB expression Bax expression	- - - + +	- + + + +	+ + + + +	+ + + + +

**Table 2:** The neurological deficits scores in experimental groups by details. This table exhibits partial scores in ten rats. Total neurological deficit scores and behavioral tests were analyzed and reported separately. (Non-parametric Kruskal-Wallis analysis).

**Groups**	**Rat**	**Raise the Tail**	**Motor Function**	**Sensory Function**	**Beam Test**	**Reflex Activity**	**Sum**	**Average**
Sham	1	1	1	2	1	1	6	5
	2	1	0	1	2	0	4	
	3	1	1	2	2	1	7	
	4	0	2	0	3	0	5	
	5	1	1	0	2	0	4	
	6	2	0	1	3	1	7	
	7	0	2	1	1	0	4	
	8	1	1	2	0	0	4	
	9	0	1	2	1	0	4	
	10	1	1	0	2	1	5	
Ischemia	1	2	2	3	4	2	13	13.5
	2	2	3	3	6	2	16	
	3	1	3	3	4	2	13	
	4	3	3	3	2	3	14	
	5	2	3	0	5	2	12	
	6	2	3	3	2	3	13	
	7	2	3	1	5	3	14	
	8	2	2	3	4	2	13	
	9	2	2	3	2	3	12	
	10	2	3	3	5	2	15	
*P-*value (Ischemia and sham)		(*P *= 0.001)	(*P < *0.001)	(*P *= 0.004)	(*P *= 0.003)	(*P *= 0.001)		
Transplant+ischemia	1	1	1	2	0	0	4	6.4
	2	1	0	0	0	0	1	
	3	2	1	3	6	2	14	
	4	1	2	0	0	0	3	
	5	2	2	2	4	0	10	
	6	0	1	3	6	3	13	
	7	1	3	0	0	1	5	
	8	1	1	3	0	0	5	
	9	1	2	3	0	0	6	
	10	1	1	1	0	0	3	
*P-*value (Transplant+ischemia and Ischemia)		(*P *= 0.002)	(*P *= 0.002)		(*P *= 0.039)	(*P *= 0.001)		

## Discussion

A significant reduction was observed in the neurological deficits, including motor, sensory, beam balance, and reflex function in the Sertoli cell-transplanted group compared with the ischemia group. These findings indicate a positive and neuroprotective effect of Sertoli cells on neurological deficits. With regard to the fact that each behavior is controlled by a specific area of the brain, it is possible that areas that have been affected by ischemia can be detected. The above-mentioned behavioral tests represent the function of the primary motor cortex, the primary and secondary somatosensory cortex, and striatum that is damaged in cerebral ischemia. So there is the hypothesis that the reduction of behavior deficits following the injection of Sertoli cells may be a reflection of a decrease in infarction of these areas. In 2001 the neuroprotective effect of IGF (growth factor that releases of Sertoli cell) on neurological deficits in stroke was examined and the reduction of neurological deficits was reported (22). GDNF and FGF were identified as effective factors of Sertoli cell in reducing neurological deficits (23-25). In 2005, the positive effect of VEGF injection in reducing neurological defects of the penumbra region was also demonstrated after the induction of the MCAO model (26). In 2008, the TGF factor showed the improved motor and sensory function in an animal model of stroke (27). Probably, Sertoli cell ameliorates the neurological deficits via the release of growth factors and infarction in related brain areas.

In this study, it was also observed that the infarct volume in the cortex and striatum decreased significantly compared with the ischemia group. This exhibits the beneficial function of the Sertoli cells in providing suitable conditions for the survival of the neurons and preventing cell death in the penumbra region, and subsequently reducing the volume of the infarction. Among all the factors secreted from Sertoli cells that have a direct effect on the reduction of infarct volume, various growth factors can be mentioned. Growth factors allow the cells to grow and survive while protecting the brain from ischemia-induced damage (28). TGF growth factor protects the neurons against excitotoxicity and cell death. TGF inhibits cellular death through inhibiting the caspase 1 and 3 signaling pathway and stimulates neurogenesis (23, 27 and 29). In some cases, TGF has a vasodilating role that increases the blood supply to the brain (30). Fibroblast growth factor, in addition to glial cells, feeds neurons and endothelial cells in the nervous system (31). FGF is an angiogenesis stimulator and a vasodilator that increases the blood flow to the brain (24), also has an important and indirect effect on reducing the size of the infarction by cytotoxicity inhibition, reduction of apoptotic Bax protein expression, and increase in Bcl-2 expression (31-33). IGF factor induces a mitotic response in neurons and also attenuates the infarct volume with anti-apoptotic effects (34-36). Eventually, the VEGF factor, which is associated with enhanced vascular density in the injured area, somewhat compensates for disturbance of blood flow and plays a significant role in the rescue of the penumbra and reducing the infarct volume (26). The previous research showed that the stimulation of mRNA GNDF expression reduces the infarct volume in an animal model of cerebral ischemia (37). It was also found that the expression rate of this factor in oligodendrocytes and macrophages of the penumbra region increased significantly (30). So, Sertoli cell probably reduced the infarction through the secretion of the growth factors.

Based on the results of this study, the blood-brain barrier in the cortex and striatum area of the transplant recipient group showed more integrity in comparison with the ischemia group. During ischemia and oxidative stress caused by reperfusion, a phenomenon called excitotoxicity occurs as a result of glutamate accumulation outside the cell and excessive entry of calcium into the neuron (38, 39). The damage of excitotoxicity in the mitochondria exerts an increase in the production of free radicals such as reactive oxygen species (ROS) and reactive nitrogen species, which activates the matrix metalloproteinase (MMPs) factor (40-42). This factor will ultimately lead to the opening of the connections between endothelial cells, the loss of integrity of the blood-brain barrier (42). Based on the mentioned information, increasing the activity of antioxidant enzymes leads to decreasing the activity of free radicals (13). In the meanwhile, Sertoli cells secrete high levels of antioxidant enzymes such as SOD, GST, GR, and GPx to protect themselves from oxidative stress (43). SOD is the first defense against free radicals of oxygen and is involved in converting superoxide anions into hydrogen peroxide. The GPx also converts hydrogen peroxide into H_2_O and O_2_. In this reaction glutathione is used as oxygen donators (44). Combining these enzymes with GST and GR effectively protects cells from oxidative stress, oxygen and hydrogen radicals, and the other oxides. Therefore, the ability of Sertoli cells to inhibit free radicals and thus to prevent the blood-brain barrier breakdown is expected (12).

It is noteworthy that our observations on brain water content have shown a reduction of the brain edema in the striatum and cortex of the transplant+ischemia group compared to the ischemia group. In fact, after reducing the damage to the blood-brain barrier, the reduction of vasogenic edema is expectable, and the results obtained in this study confirmed this hypothesis. Edema is a dangerous clinical consequence of stroke (45). Meanwhile, antioxidant enzymes secreted from Sertoli cells that prevent the breakdown of the blood-brain barrier play an important role in reducing edema and brain damage. Also, GDNF is one of the known factors in controlling brain edema. This factor indirectly reduces the blood-brain barrier permeability and consequently edema by inhibiting the pathway of caspase and inflammatory factors (23, 30).

In the present study, it was observed that NF-kB inflammatory factor expression in the cortical and striatal regions of the transplant group was significantly diminished, while expression of this factor was increased in the ischemic group resulting in severe inflammation. NF-kB is known as a nuclear factor for triggering the pathway of inflammation in the cell. This factor is activated by stimuli such as interleukin 1- beta, tumor necrosis factor (TNF-α), and ROS, and induces pathways of inflammation, immune response, cell proliferation, and apoptosis. According to available evidence, the complex role of NF-kB due to its interaction with other factors is still unclear (46). Adenosine is an intrinsic anti-inflammatory factor, which is significantly released from cells during ischemia and plays an important role in inhibiting neutrophils and reducing the inflammation as well as free radicals and endothelial cell adhesion molecules (47). On the other hand, antioxidant enzymes, especially SOD, decrease the number of free radicals such as ROS in ischemic conditions, which inhibits NF-kB (47-49). The inhibition of the NF-kB activity leads to an increase in the expression of the adenosine type A1 receptors. Increasing these receptors and increasing adenosine uptake will ultimately produce more antioxidants in the cell (50, 51). According to the results observed in this study, the effect of antioxidants released from Sertoli cells on inflammation of the brain tissue during ischemia is probable. 

Based on the present results, the transplantation of Sertoli cells in the ischemic brain of the rats reduced the expression of the Bax factor. Ischemia increases the number of free radicals that induce the internal pathway of apoptosis and increases the expression of Bax. The pro-apoptotic factor Bax is activated in the pathway of mitochondrial apoptosis by binding to the P53 tumor suppressor and Bcl-2. By increase in permeability of the mitochondrial apoptosis-induced channel, membrane pores in the outer membrane of the mitochondria, cytochrome C and other pro-apoptotic proteins from the mitochondria are released and eventually activate caspase 3 (the last apoptotic chain factor) (8, 52). On the other hand, activated mitogen-activated protein kinase (MAP Kinases) activates of pro-apoptotic factors following impairment of mitochondrial function in cerebral ischemia (53). Various growth factors released from Sertoli cells in the ischemic brain have a dual function. Neuroprotective effects of these factors are related to their delayed function; *i.e.* 6 h up to several weeks after ischemia. While in the rapid and acute phase of ischemia, most of the neurodegenerative effects are related to the cytokine activity of these factors, including the breakdown of the blood-brain barrier, edema, and inflammation (54, 55). FGF, by inhibiting MAP Kinases, reduces the expression of pro-apoptotic proteins such as Bax. It also moderates the uncontrolled entry of calcium into cells and attenuates excitotoxicity. Subsequently, it protects the neurons against death (53, 55). VEGF factor decreases the expression of Bax factor through activating proteins of cell survival pathway, such as Akt, which is an inhibitor Bad pro-apoptotic protein that inhibits the release of cytochrome C from mitochondria and thus apoptosis (54, 56). IGF also plays an important role in the suppression of Bax’s function by activating the AKT signaling pathway (57, 58). Meanwhile, the role of antioxidants in reducing the apoptosis by inhibiting ROS radicals is negligence. So, a significant reduction in expression of Bax in the striatum region of the transplant recipient group compared to the ischemia group in this study is justifiable by the optimum performance of various growth factors and antioxidant enzymes derived from Sertoli cells.

A week after the cultivation, Sertoli cells have reached a large number. To estimate the number of the cells, the expression of GATA4 as a marker of Sertoli cells was investigated by immunocytochemistry. Flow cytometry was also used to evaluate the expression of vimentin. Flow cytometry showed a purity of 83.6% of Sertoli cell (expression rate). According to the previous finding, the expression rate of Sertoli cells reported the same (59). Furthermore, the other studies reported the purity of Sertoli cell 95% and 80% in the culture, respectively (60, 61). By using morphologic analyses or staining for vimentin, purity of Sertoli cell was determined (60). Also, the purity of isolated cells (Sertoli cell) was immunostained with anti-vimentin, anit-WT1, and anti-TRA98 antibodies, which indicated >95% (62). The other research exhibited the purity of Sertoli cell more than 97% by flow cytometry with FSH receptor antibody (63).

So, according to the results of several types of research about the treatment of some neurodegenerative diseases such as Parkinson (9,65), Huntington (64), and diabetes (10, 11) with Sertoli cells, the reduction of brain damage can be expected through a pre-treatment approach with Sertoli cells.

Finally, the characteristics of Sertoli cells such as secretion of cytoprotective growth, antioxidant enzymes, and immunosuppressive mechanisms and based on the present results, it should be noted that these cells have the special ability in protecting the function of the other cells especially ischemic neurons and surrounding area.

## Conclusion

According to the results of the present study and other studies, it can be stated that the positive effects of Sertoli cell transplantation cause reduction of the ischemic damages, including decreased infarction, the blood-brain barrier permeability, brain edema and also improvement of motor function. This is likely to observed improvements in ischemic damages following the Sertoli cell transplantation, partly through inhibition of inflammatory and apoptotic factors. Ultimately, the transplant of this cell group can be an effective approach to protect the nervous system of the people susceptible to stroke.
